# Α-fetoprotein producing hepatoid gastric adenocarcinoma with neuroendocrine differentiation: A case report

**DOI:** 10.1097/MD.0000000000012359

**Published:** 2018-09-14

**Authors:** Tao Li, Tongjun Liu, Min Wang, Mingwei Zhang

**Affiliations:** Department of Colorectal and Anal Surgery, The Second Hospital of Jilin University, Changchun, China.

**Keywords:** hepatoid gastric adenocarcinoma, neuroendocrine differentiation, α-fetoprotein

## Abstract

**Rationale:**

Hepatoid gastric adenocarcinoma is a rare type of gastric cancer. The phenomenon of neuroendocrine differentiation (NED) in gastrointestinal tumor needs further research. Both hepatoid adenocarcinoma and neuroendocrine differentiation are the factors leading to a poor prognosis of gastric cancer. However, there is still no specific treatment.

**Patient Concerns:**

A 60-year-old man who had a pain and distention in his upper abdomen presented melena. Gastroscopy and pathology revealed a gastric cancer.

**Diagnoses:**

Postoperative pathology revealed a hepatoid gastric adenocarcinoma. Immunohistochemical analysis showed a-fetoprotein (AFP), hepatocyte, synaptophysin (Syn), and chromogranin A (CgA) positive, and Ki67 60% positive. A-fetoprotein producing hepatoid gastric adenocarcinoma with NED is diagnosed.

**Interventions:**

The patient was treated with an R2 radical gastrectomy, but refused chemotherapy.

**Outcomes:**

The AFP level was >2000 ng/mL (0–8.78) half a year after the surgery. There was no obvious abnormality from computed tomography (CT). The patient refused positron emission tomography computed tomography (PET-CT) and left the hospital.

**Lessons:**

Hepatoid adenocarcinoma and neuroendocrine differentiation are the factors leading to a poor prognosis of gastric cancer. It relapses easily. Long-term follow-up and regular examinations are necessary to detect relapses.

## 1 Introduction

Hepatoid gastric adenocarcinoma is a rare type of gastric cancer, which accounts for ≤1% in all gastric cancer.^[1,2]^ It is similar to hepatocellular carcinoma, and may produce α-fetoprotein (AFP). However, some patients present a normal AFP level, and some AFP producing gastric cancer is not hepatoid adenocarcinoma. The 2010 World Health Organization presented the classification of digestive system tumor.^[3]^ It included the description of neuroendocrine neoplasm in gastrointestinal tract but did not account for the phenomenon of neuroendocrine differentiation (NED). According to the reports and studies, both hepatoid adenocarcinoma and neuroendocrine differentiation are the factors leading to a poor prognosis of gastric cancer. However, there is still no specific treatment.^[1,2,4–8]^ The relevant fields need further research. Here, we present a case of AFP producing hepatoid gastric adenocarcinoma with neuroendocrine differentiation and summarize the relevant literature.

## 2 Case report

**Figure 1:**
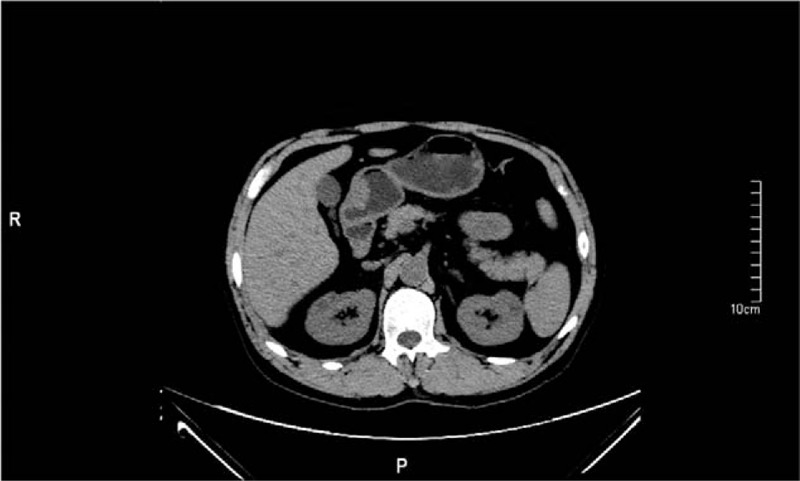
Iconography findings. Abdominal CT scan showed that there is a thickening in the front wall of the gastric antrum. CT = computed tomography.

**Figure 2:**
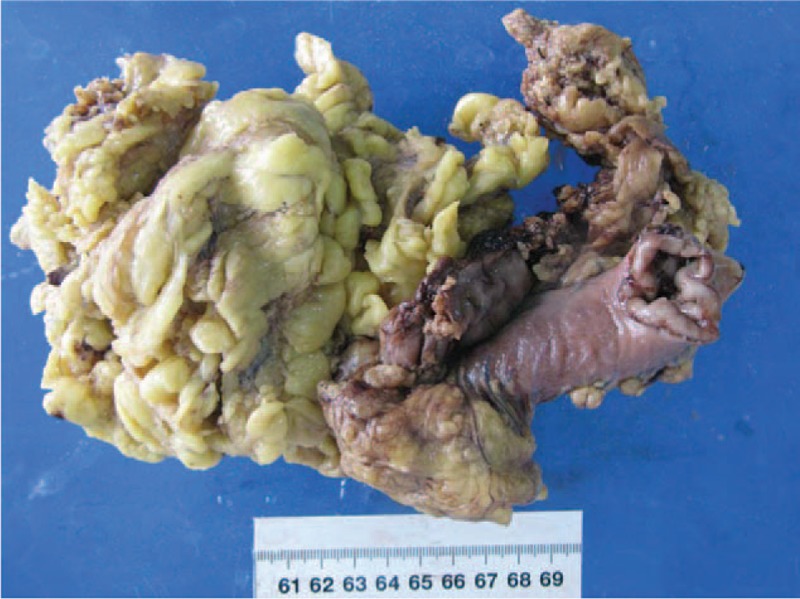
The pathological gross specimen.

**Figure 3:**
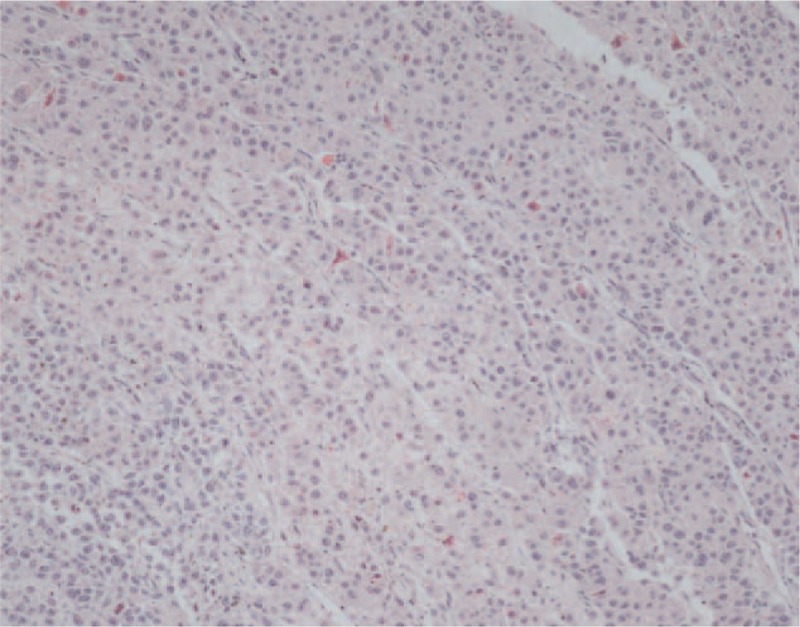
Histopathological findings. Postoperative pathology revealed a hepatoid gastric adenocarcinoma (hematoxylin and eosin stain, ×100).

**Figure 4:**
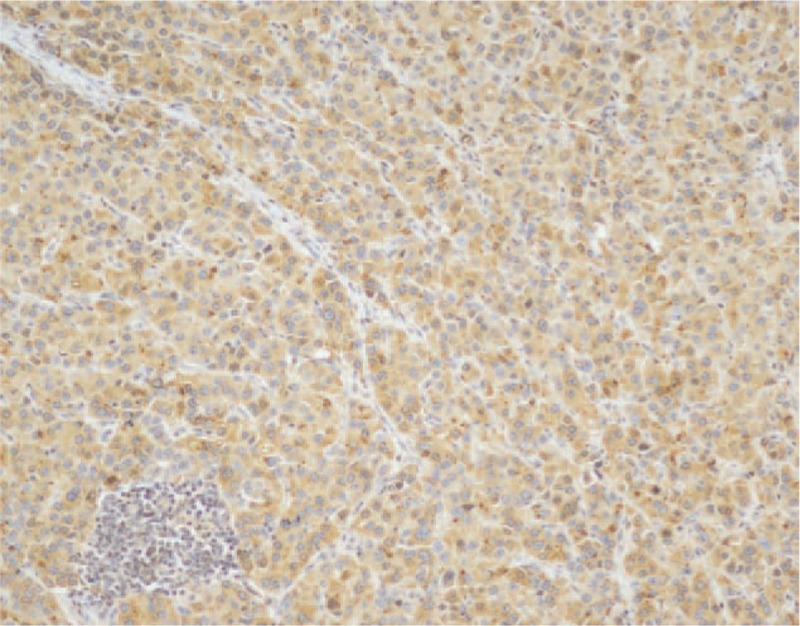
Immunohistochemical findings. Immunohistochemical analysis revealed AFP positive (immunohistochemical stain, ×100). AFP = α-fetoprotein.

**Figure 5:**
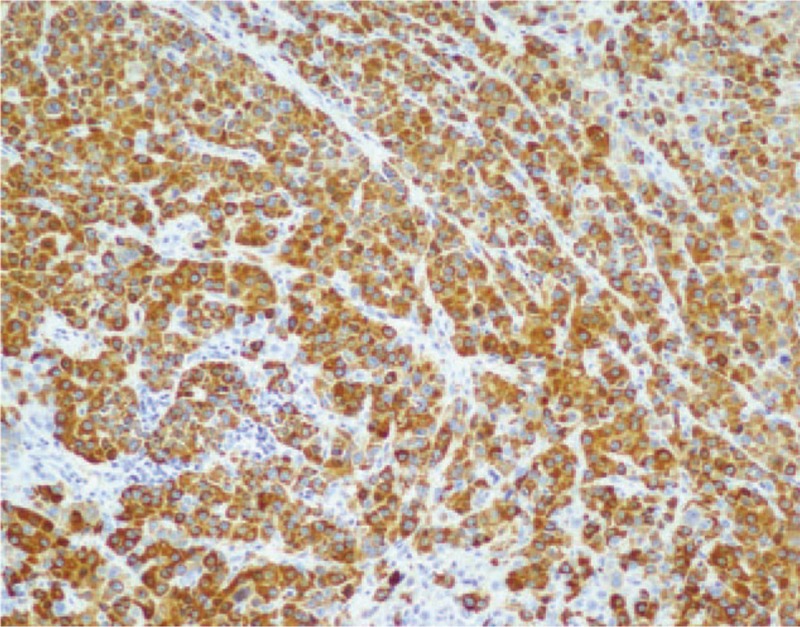
Immunohistochemical findings. Immunohistochemical analysis revealed Hepatocyte positive (immunohistochemical stain, ×100).

**Figure 6:**
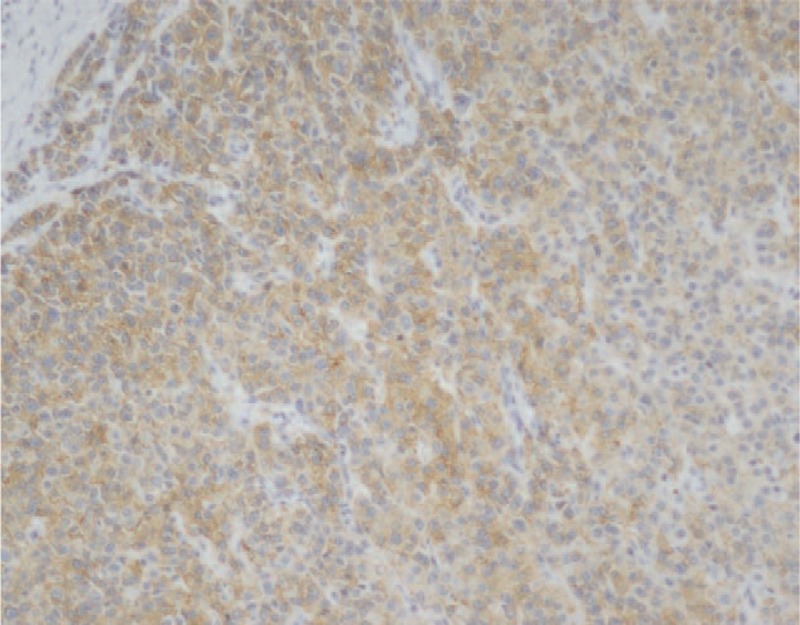
Immunohistochemical findings. Immunohistochemical analysis revealed Syn positive (immunohistochemical stain, ×100). Syn = synaptophysin.

**Figure 7:**
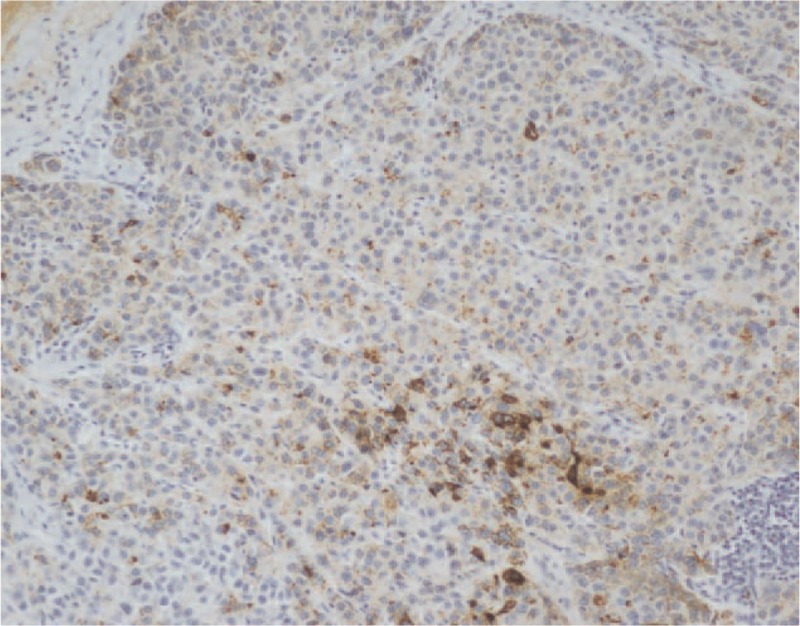
Immunohistochemical findings. Immunohistochemical analysis revealed CgA positive (immunohistochemical stain, ×100). CgA = chromogranin A.

**Figure 8:**
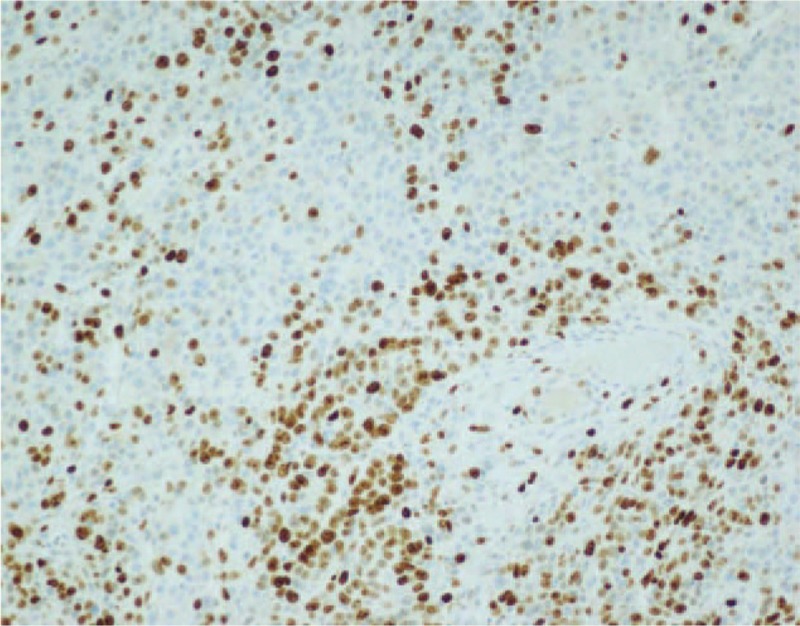
Immunohistochemical findings. Immunohistochemical analysis revealed Ki67 positive (immunohistochemical stain, ×100).

A 60-year-old man underwent a gastroscopy in a local hospital due to a pain and distention in his upper abdomen and melena for 15 days. Gastroscopy revealed a mass measured 3.5 cm diameter on the front wall of the gastric antrum. The lesser curvature and prepyloric region were involved. It had an irregular surface and a large and deep ulcer in the center. The pathologic diagnosis was a poorly differentiated adenocarcinoma. He then came to our hospital for further treatment. Further examination revealed that the AFP level was 1683.33 ng/mL (<9.0). Abdominal computed tomography (CT) revealed a thickening in the front wall of the gastric antrum (Fig. 1). The radial line was about 16 mm. There was no obvious abnormality in the liver, except multiple cysts. The patient was treated with R2 radical gastrectomy. Postoperative pathology revealed a hepatoid gastric adenocarcinoma with NED (Figs. 2 and 3). It was invading the muscular layer of the stomach. The tumor could be found in the vessel but not in the epiploon and incisal edge. One lymph node was positive on the greater curvature (1/11), and all were negative on the lesser curvature (0/9). TNM staging was T_2_N_1_M_x_. Immunohistochemical analysis showed AFP, Hepatocyte, synaptophysin (Syn) and chromogranin A (CgA) positive, and Ki67 60% positive (Figs. 4–8).

The AFP level was 189.98 ng/mL (<9.0) 10 days after the surgery. The patient refused chemotherapy and left the hospital 12 days after the surgery. The patient came back to the hospital half a year after the surgery. The AFP level was >2000 ng/mL (0–8.78). There was no obvious abnormality from CT. The patient refused positron emission tomography computed tomography (PET-CT) and left the hospital.

## 3 Discussion

Hepatoid gastric adenocarcinoma is a rare pathological type which accounts for ≤1% in all gastric cancer.^[1,2]^ It has a similar morphological feature with hepatocellular carcinoma in the pathological examination. It has been known that not all hepatoid gastric adenocarcinoma produce AFP, some patients express negative.^[1]^ Although hepatoid adenocarcinoma is the most common, there are some other types of AFP-producing gastric cancer, such as yolk sac tumor-like and fetal gastrointestinal type.^[1,9]^ And in some AFP producing cases, the component of the tumor is not unique.^[10]^ According to the literature, the incidence of AFP producing gastric cancer is 1.3% to 15%.^[10]^ Above all, there is some clinical experience. On one hand, gastric cancer without AFP producing cannot exclude the possibility of hepatoid adenocarcinoma. On the other, AFP producing gastric cancer may not hepatoid adenocarcinoma. The diagnosis of hepatoid gastric adenocarcinoma still needs pathology.

Patients with hepatoid gastric adenocarcinoma usually have had the liver metastases when coming to the hospital. However, it is difficult to distinguish if the tumor in the liver is primary or transferred. The examination of Sal-like protein 4 (SALL4) is considered a valid method to differentiate them.^[1]^ It is positive in hepatoid gastric adenocarcinoma but negative in hepatocellular carcinoma. Currently, there are various researches on the abnormal expression of SALL4. SALL4 is considered an important marker to assess the prognosis of malignancies.^[11]^

On imaging, the majority of hepatoid gastric adenocarcinoma patients will be found a liver metastasis. It presents a multifocal hepatocellular carcinoma or with a rich vascular transfer.^[12]^ Lee et al had a study on CT features of hepatoid gastric adenocarcinoma. Besides an abnormal thickening of the gastric wall, it performs obviously to transfer to the liver and lymph nodes, and primary and metastatic tumor gets a venous invasion surrounded easily.^[13]^

Hepatoid gastric adenocarcinoma does not have specific symptoms. It presents similar clinical manifestations with general gastric cancer. However, patient's conditions are progressing rapidly. Liver and lymph nodes metastases are usually found when patients coming to the hospital. This is the main cause that leads to a poor prognosis.^[1,4,12]^ Currently, radical excision is the main treatment. However, although the radical resection is successful, recurrence probably occurs in a short term. The AFP of our patient had an obvious descent after surgery, but was in an extremely high level when returning to the hospital after half a year. The patient probably had had a recurrence. There is no specific chemotherapy regimen for hepatoid gastric adenocarcinoma at present.^[5]^ Liu et al reported a prognostic study which included hepatoid gastric adenocarcinoma, other types of AFP producing gastric cancer and AFP negative gastric cancer. The 1-year survival rates were 30%, 64%, and 95%, the 3-year were 13%, 47%, and 57%, and the 5-year were 9%, 41%, and 38% (*P* < .01).^[14]^ It indicates that the prognosis of AFP producing is poorer than AFP negative. Moreover, the identification of hepatoid adenocarcinoma is essential, for it has the poorest prognosis. Qu et al did a research on hepatoid gastric adenocarcinoma patients from China. It showed that the 3-year survival rate was 7.36%, the median survival time was 10 months, and the survival condition was obviously influenced by metastases. Furthermore, hepatoid gastric adenocarcinoma is more common in old male, and often to be ulcerated occurring in the gastric antrum.^[6]^

Immunohistochemical analysis in our patient showed that CgA and Syn were positive. It means that the patient had an AFP producing hepatoid gastric adenocarcinoma with NED. The classification of digestive system tumor from 2010 World Health Organization accounts for neuroendocrine neoplasm. The diagnosis depends on immunohistochemical analysis that if CgA and Syn are positive. Neuroendocrine neoplasm can be divided into 3 grades including G1, G2, and G3, depending on Ki67. On pathology, it can be split into well-differentiated neuroendocrine tumor (NET) including grade G1 and G2, and poorly differentiated neuroendocrine carcinoma (NEC) including grade G3.^[3,15]^ Neuroendocrine neoplasm from alimentary tract is uncommon. It only accounts for 1% in the esophagus, 0.1% to 0.4% in the stomach, and 0.2% in the colon.^[16,17]^ Moreover, there is still a specific NEC which is named mixed adenoneuroendocrine carcinoma (MANEC). On morphology, it has both adenocarcinoma and neuroendocrine carcinoma, and each component accounts for at least 30%.^[3,18]^ MANEC gastric cancer is extremely rare and the histological origin is still unclear.^[16]^ MANEC that is different from NED has definite diagnostic criteria. It should be distinguished in the clinical treatment. However, the classification of digestive system tumor in 2010 World Health Organization did not give a detailed description on adenocarcinoma with NED.

Currently, the conception of adenocarcinoma with NED is widely used in relevant studies. We may find this phenomenon in many tissues and organs including breast, lung, colorectum, gallbladder, prostate, ovary, and so on.^[19–24]^ There is also some research on gastric cancer with NED.

Melissari et al^[25]^ reported that the phenomenon of gastric cancer with NED has no relation to the gender, age, histological type, and tumor stage. Zhang et al hold the view that compared with gastric cancer without NED, gastric cancer with NED has a lower 1 and 3-year survival rates. It also has an obviously shorter overall survival time. NED may come to be an important indicator to assess the prognosis.^[7]^ Similarly, Naritomi et al had a study on 337 gastric adenocarcinoma and found that endocrine cell positive group had a higher incidence of lymph nodes metastasis than negative when submucosa or muscularis propria invaded. Moreover, it also led to a poorer prognosis. The expression of endocrine cell may be a prognostic factor.^[26]^

The main treatment of gastric cancer with NED is surgery. However, there is still no specific treatment criterion. The sensitivity of chemotherapy also needs further research. Thus, this area still needs more data.^[7,8]^ The reports and studies of hepatoid gastric adenocarcinoma with NED are rare. Wincewicz et al^[27]^ reported a case of hepatoid gastric adenocarcinoma with NED and osteoclast-like giant cells.

From the literature review, both hepatoid adenocarcinoma and NED are poor prognostic factors for gastric cancer. However, both of them still have no specific treatment criterion and need more reports and research. The 2 factors are what we should take care of on the clinical work.

## Author contributions

**Conceptualization:** Tao Li, Mingwei Zhang.

**Data curation:** Tao Li, Min Wang.

**Writing – original draft:** Tao Li.

**Writing – review & editing:** Tao Li, Tongjun Liu, Mingwei Zhang.
